# Body mass index, cognitive deficit and depressive symptoms in high cardiovascular risk patients

**DOI:** 10.1590/S1980-57642010DN40400010

**Published:** 2010

**Authors:** Amanda Lucas da Costa, Juliana Santos Varela, Matheus Roriz Cruz, Andry Fitterman Costa, Paulo Dornelles Picon, Emilio Moriguch, Márcia L.F. Chaves

**Affiliations:** 1 Dementia Clinic, Neurology Service, Hospital de Clínicas de Porto Alegre, Porto Alegre RS, Brazil.; 2 Medical Sciences Post-Graduation Course, UFRGS School of Medicine, Porto Alegre RS, Brazil.; 3 Dyslipidemia and High Cardiovascular Risk Clinic, Hospital de Clínicas de Porto Alegre, Porto Alegre RS, Brazil.

**Keywords:** obesity, cognitive impairment, depression

## Abstract

To evaluate the relationship of obesity, cognitive impairment and depressive symptoms in patients with high cardiovascular risk.

**Methods:**

A sample of 93 patients aged 50 years or older was selected from the Center of Dyslipidemia and High Cardiovascular Risk from Hospital de Clínicas de Porto Alegre (HCPA). Patients with stroke were excluded. For cognitive evaluation, the MMSE (Mini Mental State Examination) was used. A score of 24 or less was considered as cognitive impairment, and for those who had 4 years or less of education, the cutoff point was 17. The GDS-15 (Geriatric Depression Scale) was also used, with the cutoff of 6 for presence of depressive symptoms.

**Results:**

Obese patients showed lower mean MMSE scores compared to non-obese patients (p=0.0012). Additionally, for every one point increase in BMI above 30 there was a 27% increase in the chances of the patient having cognitive impairment. The obese patients presented 31% chance of having cognitive impairment compared with overweight subjects.

**Conclusions:**

Our findings corroborated the association between obesity and cognitive impairment in high cardiovascular risk patients. This association however, was not observed for depressive symptoms.

## Abstract (pt)

Avaliar a associação entre obesidade, comprometimento cognitivo e sintomas depressivos em pacientes de alto risco cardiovascular.

**Métodos:**

Foi selecionada uma amostra de 93 pacientes com 50 anos ou mais em acompanhamento no Centro de Dislipidemia e Alto Risco Cardiovascular do Hospital de Clínicas de Porto Alegre (HCPA). Pacientes com história de acidente vascular cerebral (AVC) foram excluídos. Para a avaliação cognitiva, foi utilizado o MEEM (Mini Exame do Estado Mental). Escores de 24 ou menos foram considerados como comprometimento cognitivo, para pacientes com 4 ou menos anos de escolaridade, o ponto de corte usado foi de 17 pontos. A escala GDS-15 (Geriatric Depression Scale) também foi utilizada, sendo adotado o ponto de corte 6 para a presença de sintomas depressivos.

**Resultados:**

Pacientes obesos apresentaram valores menores na média dos escores do MEEM quando comparados a pacientes não-obesos (p=0,0012). Além disso, para cada ponto de acréscimo no IMC acima de 30, houve um aumento de 27% na chance do paciente ter comprometimento cognitivo. Os pacientes obesos apresentaram 31% de chance de ter comprometimento cognitivo quando comparados com pacientes com sobrepeso.

**Conclusões:**

Os achados do presente estudo corroboram a associação entre obesidade e comprometimento cognitivo em pacientes de alto risco cardiovascular. Entretanto, esta associação não foi observada ao se analisar os sintomas depressivos.

The prevalence of overweight and obesity exceeds 50% among adults in the U.S. and Europe, and is even higher among women aged 50 years or older. Research has shown that around 72% of older Americans (≥60 years) presented overweight or obesity, and more than 35% were obese.^1^ However, this has been changing, where men and children are also showing a high prevalence of obesity and overweight.^2^ According to data from the Brazilian Ministry of Health, obesity and overweight have shown the same increasing trend among Brazilian adults, rising from 11.4% in 2007 to 13% in 2008. In Brazil, obesity is higher among women (13.6%) than men (12.4%). In the age group of 65 years or older, obesity reached 20.5% among women and 11.2% among men. The frequency of overweight among Brazilians aged 18 years or more was 43.3%. Men showed a higher frequency of overweight (47.3%) than women (39.5%).^3^

Obesity increases the risk of cardiovascular diseases and is related to other comorbidities such as depression and diabetes.^4^ The increasing number of people with overweight and obesity threatens a high impact on costs for the public health system.

Recently, obesity and overweight have been associated to the risk of developing dementia, which is very important in populations where the older strata are increasing.^5,6^ Dementia, especially of vascular etiology, is associated with cardiovascular complications, and recent studies have shown that Alzheimer’s disease can result from cardiovascular factors.^7^ One group of factors is body fat (represented by the BMI), obesity (BMI >30kg/m^2^) and central obesity (measured by waist circumference).^1^

Although dementia affects 6 to 10% of individuals aged 65 years or more, its association with obesity is not yet fully understood. Obesity is an important risk factor for hyperinsulinism and diabetes mellitus, and both are risk factors for dementia. However, results from studies exploring the association of obesity and dementia are conflicting.^8-11^

The adipose tissue has been traditionally considered a passive tissue – an “energy depot”.^12^ However, recent evidence has revealed that the tissue is active and produces a series of important substances for body function (adipokines) and inflammatory cytokines (TNF-α and IL-6).^13^ These products may potentially impact cognition and aging, since they affect hypothalamic functions and learning processes. Leptin was found to be inversely correlated with the volume of gray matter in brain regions of obese subjects compared to slim subjects.^6,12,14,15^

Hyperinsulinemia can also have some influence on the mechanism that links dementia to adiposity. Insulin can cross the blood-brain barrier and compete with the β-amyloid for the enzyme that degrades insulin in the brain (insulin degrading enzyme – IDE).^7^ Since it can also be produced in the brain, insulin can have a beneficial effect in β-amyloid clearance.^16^ Peripheral hyperinsulinemia can also inhibit the production of insulin in the brain and impair amyloid clearance, increasing the risk of developing Alzheimer disease.^16^ Advanced glycation end products (AGEs), which result from intolerance to glucose and from diabetes, and frequently present together with adiposity, are responsible for the damage to target organs and can be differentiated in senile plaques and neurofibrillary tangles by immunohistochemistry. Moreover, it was noted that receptors for AGEs are specific for amyloids, and this could potentially facilitate neural damage.^6^

Depression is one of the most frequent causes of mental suffering and worsening of life quality in elderly individuals.^17^ The prevalence of depressive symptoms in the population aged 65 years or more in the community ranges from 10.3% to 13.5%^18^, and in Brazil this figure reaches 14.3%. Elderly people are more susceptible to depression since they have reduced social perspectives, decline in general health, frequent losses, biological, vascular, structural and functional changes, in addition to the neuroendocrinological and neurochemical dysfunctions that occur in the brain as aging progresses.^19,20^ Thus, the development of depression in the elderly has a multifactorial origin.

Since depression and dementia are the most frequent mental health problems in the elderly, these disorders commonly coexist, suggesting a strong association between them. This may lead to severe consequences such as worsening of life quality and functional decline, increasing morbidity and mortality as well as the need for healthcare assistance.^21^ The association between dementia and depression is also important. Depressed elderly individuals may show cognitive impairment, and higher risk of developing dementia.^22^

In this context, identifying risk factors for dementia and depression is necessary. Several findings have suggested an association between cardiovascular risk factors and the pathogenesis of Alzheimer’s disease. These findings include higher density of cortical senile plaques in patients without dementia but with severe coronary artery disease,^23^ positive significant association between atherosclerotic index and AD diagnosis;^24^ and positive association between high homocystein levels and AD prevalence.^22^ The risk of developing dementia is higher in patients with pathological conditions associated to higher levels of cholesterol, as in cardiovascular disease and atherosclerosis.^5^ Other risk factors frequently associated with dementia are stroke, hypertension, presence of the apolipoprotein E^4^ allele, dyslipidemia, and hyperinsulinemia.^21,22,24^ Systemic arterial hypertension, diabetes, hypercholesterolemia and coronary or carotid artery disease can lead to cerebrovascular disease, hypoperfusion and ischemia.^12^ In cases where alterations occurred in areas responsible for cognitive functions, dementia sometimes developed.^25^

Given the importance of obesity as a potential risk factor for dementia and depressive symptoms, the main goal of this study was to evaluate the association among obesity, depressive symptoms and cognitive deficit in patients aged 50 years or older followed in a high cardiovascular risk center.

## Methods

A cross-sectional study was conducted on a sample of patients from the Dyslipidemia and High Cardiovascular Clinic, from Hospital de Clínicas de Porto Alegre (HCPA), southern Brazil. Ninety-three consecutive patients seen from January 2005 onwards were included. All patients with clinical or documented stroke were excluded. Gender, age, years of education and cardiovascular risk factors are shown in Table 1.

**Table 1:** Demographic and clinical data of obese and non-obese groups.

Variables	Non-obese group (n=58)	Obese group (n=34)	P value
Age (years)	65.2±6.9	61.8±7.6	0.027
Gender Male Female	25 (43.10%) 33 (56.90%)	6 (17.65%) 28 (82.35%)	
Family income (minimum wages*)	19.0±5.1	17.5±5.1	0.197
Education (years) 0 0-8 years 8-11 years >11 years	5 (5.43%) 34 (36,96%) 9 (9.78%) 7 (7.60%)	4 (4.35%) 22 (23.91%) 7 (7.60%) 0 (0.00%)	0.419
MMSE	26±4.5	24±5.0	0.105
GDS	5.8±3.8	4.0±3.1	0.022
Smoking habit Currently Never In the past	7 (7.60%) 26 (28.26%) 24 (26.08%)	2 (2.17%) 19 (20.65%) 13 (14.13%)	0.444
Alcohol use Currently In the past Never used	8 (8.70%) 9 (9.78%) 38 (41.30%)	3 (3.26%) 5 (5.43%) 26 (28.26%)	0.444
Diabetes mellitus	16 (17.39%)	14 (15.21%)	0.451
Coronary artery disease	22 (23.91%)	12 (13.04%)	0.451
Systemic arterial hypertension	45 (48.91%)	32 (34.78%)	0.451
Cardiac failure	4 (4.35%)	1 (1.08%)	0.451
Depressive symptoms	6 (6.52%)	5 (5.43%)	0.451

* 1 minimum wage=US$ 300.

A detailed clinical history with an emphasis on past or recent cardiovascular events including the need for emergency medical assistance or hospital stay; follow-up of non-pharmacological interventions (cessation of smoking, weight loss, healthy diet, reduced salt ingestion, physical exercises, among others) and pharmacological interventions and their adequate use, and a physical examination was performed in all patients evaluated at the High Cardiovascular Risk Center. Blood assays assessing Total cholesterol, HDL cholesterol, LDL cholesterol, triglycerides, glucose, ALT, AST, GGTP, electrolytes, creatinine, uric acid and TSH were performed. Definition of high cardiovascular risk was LDL cholesterol >100 mg/dL and any evidence of atherosclerotic disease or diabetes mellitus plus other cardiovascular risk, and included those participants with LDL cholesterol >130 mg/dL or triglycerides >200 mg/dL. All patients were considered as having a high cardiovascular risk. Patients were not performing regular physical activity.

The Mini Mental State Examination (MMSE) was used to screen for cognitive function.^26,27^ Patients with more than 4 years of education that scored less than 24 points, and patients with 4 or less years of education that scored less than 17 points, were considered as having cognitive deficit. The Geriatric Depression Scale was used to evaluate depressive symptoms (GDS-15).^28^ Patients with scores of 6 and above were considered as having depressive symptoms.

The body mass index (BMI) was calculated by dividing patient weight by height squared (kg/m^2^). Patients with BMI <25 were classified as ideal weight, BMI >25 as above ideal weight, and BMI >30 as obese.

The study was approved by the Ethics Committee for Medical Research at Hospital de Clínicas de Porto Alegre. Patients signed an informed consent before being enrolled onto the study.

### Data analysis

Descriptive statistics (mean, SD and frequency) were calculated for demographic data, the MMMSE and GDS. Categorical data were analyzed with the Chi-square test with Yates correction to check association among them, and Student’s t test was employed for parametric variables. A linear regression model was used to evaluate correlations with the MMSE scores. A multivariate logistic regression model was used to evaluate associations to the outcome cognitive impairment. The statistical analysis was performed using the Statistical Package for the Social Sciences - SPSS version 11.5 for Windows. Statistical significance was defined as p<0.05.

## Results

A significant difference in MMSE scores, adjusted for age and gender, was observed between the obese group (BMI >30) and the non-obese group (p=0.0012). The obese group showed lower scores on the MMSE (Table 2). No significant difference in depressive symptoms (GDS-15 scale) was observed between groups (p=0.14) (Table 2).

**Table 2:** Comparison of MMSE and GDS, adjusted for age and gender, between obese and non-obese groups (Student's t test for independent samples).

Variables	Obese (n=34)	Non-Obese (n=58)	p value
MMSE	23.7±5.0	26.5±3.77	0.006
MMSE Adjusted	23.4±0.77	26.6±0.57	0.0012
GDS	6.0±4.0	4.3±3.2	0.040
GDS Adjusted	5.7±0.67	4.4±0.49	0.14

Using a linear regression model adjusted for age and gender, with the MMSE as the dependent variable, obesity was correlated with lower values on the MMSE (p=0.0001; B= –0.48). There was also a significant correlation of depressive symptoms with BMI (p=0.04; B=0.24).

The multivariate logistic regression analysis with the MMSE as the dependent variable (cutoff 24), adjusted for age and gender, and with BMI (cutoff 30) and depression (cutoff 6) as independent variables, showed significant association with BMI (p=0.005; OR=4.66; 95% CI 1.59-13.70). Thus, patients with BMI >30 had a 4.66-fold greater probability presenting cognitive impairment compared to patients with BMI <30. No association was observed between depressive symptoms and cognitive status (Table 3). These variables explained 74.3% of the variance. The logistic regression for the outcome obesity (BMI ≥30) with MMSE, GDS, age and education as independent variables, found only MMSE to be significantly associated with obesity (OR=3.7; 95% CI 1.08-12.67) (Table 4). The variables explained 71% of the variation.

**Table 3:** Logistic Regression analysis for MMSE outcome with obesity (BMI) and depression (GDS) as independent variables.

Variables	B	Wald	P	OR	95% CI
Obesity BMI >30	1.539	7.825	0.005	4.66	1.59-13.70
GDS Score >6	0.003	0.000	0.996	1.00	0.34-3.00
Constant	0.142	0.090	0.764		

**Table 4:** Logistic Regression analysis for obesity outcome (BMI) with MMSE and depression (GDS) as independent variables.

Variables	B	Wald	P	OR	95% CI
MMSE (cut-offs)	1.309	4.348	.037	3.703	1.082-12.673
Age	-.065	3.051	.081	.937	.872-1.008
GDS (cut-offs)	.666	1.570	.210	1.947	.687-5.523
Education	-.144	.520	.471	.866	.586-1.280
Constant	3.271	1.651	.199	26.339	--

## Discussion

The objective of the present study was to evaluate the relationship among depressive symptoms, body mass index (obesity), and cognitive performance in patients 50 years or older with high cardiovascular risk. For this purpose, the Mini Mental State Examination – MMSE – and the Geriatric Depression Scale with 15 items – GDS-15 were applied. To evaluate the impact of obesity patients were subdivided into obese and non-obese groups according to BMI (cut-off 30). Significant differences in MMSE scores, age and gender adjusted, were observed between the obese and non-obese groups. No difference in depression scores was observed between the two groups.

Obese patients were also found to be 4.66 times more likely to present cognitive impairment (MMSE below cut-off of 24). Similarly, no increased chance of cognitive impairment was observed among the depressives.

Obesity is an epidemic disorder in adults and children in developed and developing countries which presents high morbimortality.^29-31^ It is implicated in a future decline in life expectancy of the world population because it is associated with various comorbidities such as arterial hypertension, diabetes mellitus II, dyslipidemia, sleep apnea, and even some types of cancer.^18-20^

It is well known that obesity is a risk factor for cardiovascular diseases (CVD), arterial hypertension, and myocardial infarct.^29,31^ However, it has also been demonstrated that this patient group (CVD, arterial hypertension, and myocardial infarct) had a good prognosis over the long term.^18^ Many paradoxes and questions remain on this topic. Obesity has a significant impact on cardiovascular diseases: heart failure, arterial coronary disease, sudden heart death, and atrial fibrillation are all associated with reduced global survival.^17,29-31^ Despite the adverse association, paradoxical results have been observed in which individuals who were overweight and obese with cardiovascular disease and old age presented better prognosis compared to non-obese individuals.^17,32,33^ In the present study, we did not confirm any association between body mass index (obesity) and education after grouping patients into four classes of educational attainment, although this relationship has been reported.^34^ However, the distribution of patients above the cut-off of 8 years reveals that only 7.6% of the obese participants had higher education, whereas 17% of non-obese subjects were in these categories. Since most patients were in lower education groups, a further evaluation based on larger samples could clarify this issue.

Given obesity and overweight have been related to the risk of developing dementia,^6^ the findings of the present study are important to reinforce the role of these risk factors even to cognitive impairment. Dementia, vascular or Alzheimer's disease, are associated to some degree to cardiovascular factors,^19^ and obesity represents an important risk factor for cardiovascular abnormalities. The other causal relationship between obesity and dementia is through hyperinsulinism and diabetes mellitus, both risk factors for cognitive impairment and dementia.^6^

It is well known that adipocytes and inflammatory interleukins may affect other body systems. These products may potentially affect cognition and aging, for they affect hypothalamic functions and learning processes. Leptin was inversely correlated with the volume of gray matter in brain regions of obese subjects compared to slim subjects.^6,12,14,15^

Previous studies have associated high glucose levels with the inhibition of enzymes that degrade the beta-amyloid protein.

Evaluating cognition in elderly or obese patients has become essential, because dementia is a condition that currently affects around 6 million Americans and 2 million Brazilians,^35,36^ and is among the ten leading causes of death in elderly individuals over the age of 65 in developed countries.^37^

Identifying individuals with cognitive deficit is fundamental for therapeutic intervention planning, family orientation, reduction of accidents, and maybe in some cases to delay the dementia process.^38^ Depressive symptoms and dementia are the most common mental health problems in this age group and both have impact on quality of life of patients and their families.

In our study, we observed no relationship between obesity and depressive symptoms, although this is a common complaint in the population seen in our clinic probably because they have a chronic disease.

One limitation of the present study was the selection of the sample in a tertiary hospital, characterized by very specialized care. Therefore, patients presented many comorbidities, which may in turn have increased the possibility of cognitive impairment in this sample. On the other hand, this point requires further investigation in samples of patients with multiple cardiovascular pathologies (arterial systemic hypertension, coronary artery disease, obesity) because they constitute the part of the population which places the highest burden on the public health system. Patients with clinical or documented stroke were excluded. However, silent stroke was not evaluated in this sample. Silent stroke has been associated with increased C-reactive protein and interleukin-6, coronary artery disease, body mass index, and alcohol consumption, as well as overt stroke, dementia, depression, and aspiration pneumonia.^39^ The relationship of silent brain infarction, body mass index, coronary artery disease and cognitive status deserves further in-depth investigation in a Brazilian sample.

In conclusion, considering that cognitive decline, obesity and de­pressive symptoms are a worldwide problem, establishing preventive or curative treatment where available is a major health challenge, and vascular risk factors are a promising research pathway for elucidating these conditions.
